# How to use and assess qualitative research methods

**DOI:** 10.1186/s42466-020-00059-z

**Published:** 2020-05-27

**Authors:** Loraine Busetto, Wolfgang Wick, Christoph Gumbinger

**Affiliations:** 1 Department of Neurology, Heidelberg University Hospital, Im Neuenheimer Feld 400, 69120 Heidelberg, Germany; 2 Clinical Cooperation Unit Neuro-Oncology, German Cancer Research Center, Heidelberg, Germany

**Keywords:** Qualitative research, Mixed methods, Quality assessment

## Abstract

This paper aims to provide an overview of the use and assessment of qualitative research methods in the health sciences. Qualitative research can be defined as the study of the *nature* of phenomena and is especially appropriate for answering questions of *why* something is (not) observed, assessing complex multi-component interventions, and focussing on intervention improvement. The most common methods of data collection are document study, (non-) participant observations, semi-structured interviews and focus groups. For data analysis, field-notes and audio-recordings are transcribed into protocols and transcripts, and coded using qualitative data management software. Criteria such as checklists, reflexivity, sampling strategies, piloting, co-coding, member-checking and stakeholder involvement can be used to enhance and assess the quality of the research conducted. Using qualitative in addition to quantitative designs will equip us with better tools to address a greater range of research problems, and to fill in blind spots in current neurological research and practice.

## Aim

The aim of this paper is to provide an overview of qualitative research methods, including hands-on information on how they can be used, reported and assessed. This article is intended for beginning qualitative researchers in the health sciences as well as experienced quantitative researchers who wish to broaden their understanding of qualitative research.

## What is qualitative research?

Qualitative research is defined as *“the study of the nature of phenomena”,* including *“their quality, different manifestations, the context in which they appear or the perspectives from which they can be perceived”*, but excluding *“their range, frequency and place in an objectively determined chain of cause and effect”* [1]. This formal definition can be complemented with a more pragmatic rule of thumb: qualitative research generally includes data in form of *words* rather than *numbers* [2].

## Why conduct qualitative research?

Because some research questions cannot be answered using (only) quantitative methods. For example, one Australian study addressed the issue of why patients from Aboriginal communities often present late or not at all to specialist services offered by tertiary care hospitals. Using qualitative interviews with patients and staff, it found one of the most significant access barriers to be transportation problems, including some towns and communities simply not having a bus service to the hospital [3]. A quantitative study could have measured the number of patients over time or even looked at possible explanatory factors – but only those previously known or suspected to be of relevance. To *discover reasons* for observed patterns, especially the invisible or surprising ones, qualitative designs are needed.

While qualitative research is common in other fields, it is still relatively underrepresented in health services research. The latter field is more traditionally rooted in the evidence-based-medicine paradigm, as seen in "*research that involves testing the effectiveness of various strategies to achieve changes in clinical practice, preferably applying randomised controlled trial study designs (...)*" [4]. This focus on quantitative research and specifically randomised controlled trials (RCT) is visible in the idea of a hierarchy of research evidence which assumes that some research designs are objectively better than others, and that choosing a "lesser" design is only acceptable when the better ones are not practically or ethically feasible [5, 6]. Others, however, argue that an objective hierarchy does not exist, and that, instead, the research design and methods should be chosen to fit the specific research question at hand – *"questions before methods"* [2, 7–9]. This means that even when an RCT is possible, some research problems require a different design that is better suited to addressing them. Arguing in JAMA, Berwick uses the example of rapid response teams in hospitals, which he describes as "*a complex, multicomponent intervention – essentially a process of social change"* susceptible to a range of different context factors including leadership or organisation history. According to him, *"[in] such complex terrain, the RCT is an impoverished way to learn. Critics who use it as a truth standard in this context are incorrect"* [8]*.* Instead of limiting oneself to RCTs, Berwick recommends embracing a wider range of methods*,* including qualitative ones, which for *"these specific applications, (...) are not compromises in learning how to improve; they are superior"* [8].

Research problems that can be approached particularly well using qualitative methods include assessing complex multi-component interventions or systems (of change), addressing questions beyond “what works”, towards “what works for whom when, how and why”, and focussing on intervention improvement rather than accreditation [7, 9–12]. Using qualitative methods can also help shed light on the “softer” side of medical treatment. For example, while quantitative trials can measure the costs and benefits of neuro-oncological treatment in terms of survival rates or adverse effects, qualitative research can help provide a better understanding of patient or caregiver stress, visibility of illness or out-of-pocket expenses.

## How to conduct qualitative research?

**Fig. 1:**
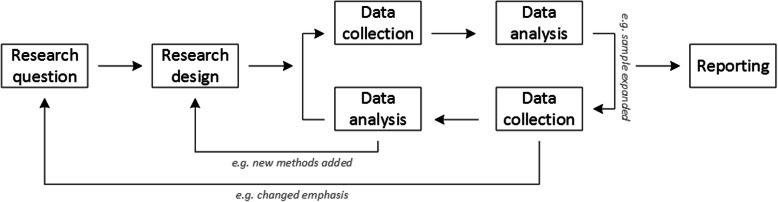
Iterative research process

Given that qualitative research is characterised by flexibility, openness and responsivity to context, the steps of data collection and analysis are not as separate and consecutive as they tend to be in quantitative research [13, 14]. As Fossey puts it*: “sampling, data collection, analysis and interpretation are related to each other in a cyclical (iterative) manner, rather than following one after another in a stepwise approach”* [15]. The researcher can make educated decisions with regard to the choice of method, how they are implemented, and to which and how many units they are applied [13]. As shown in Fig. 1, this can involve several back-and-forth steps between data collection and analysis where new insights and experiences can lead to adaption and expansion of the original plan. Some insights may also necessitate a revision of the research question and/or the research design as a whole. The process ends when saturation is achieved, i.e. when no relevant new information can be found (see also below: sampling and saturation). For reasons of transparency, it is essential for all decisions as well as the underlying reasoning to be well-documented.

While it is not always explicitly addressed, qualitative methods reflect a different underlying research paradigm than quantitative research (e.g. constructivism or interpretivism as opposed to positivism). The choice of methods can be based on the respective underlying substantive theory or theoretical framework used by the researcher [2].

### Data collection

The methods of qualitative data collection most commonly used in health research are document study, observations, semi-structured interviews and focus groups [1, 14, 16, 17].

#### Document study

Document study (also called document analysis) refers to the review by the researcher of written materials [14]. These can include personal and non-personal documents such as archives, annual reports, guidelines, policy documents, diaries or letters.

#### Observations

Observations are particularly useful to gain insights into a certain setting and actual behaviour – as opposed to reported behaviour or opinions [13]. Qualitative observations can be either *participant* or *non-participant* in nature. In participant observations, the observer is part of the observed setting, for example a nurse working in an intensive care unit [18]. In non-participant observations, the observer is “on the outside looking in”, i.e. present in but not part of the situation, trying not to influence the setting by their presence. Observations can be *planned* (e.g. for 3 h during the day or night shift) or *ad hoc *(e.g. as soon as a stroke patient arrives at the emergency room). During the observation, the observer takes notes on everything or certain pre-determined parts of what is happening around them, for example focusing on physician-patient interactions or communication between different professional groups. Written notes can be taken during or after the observations, depending on feasibility (which is usually lower during participant observations) and acceptability (e.g. when the observer is perceived to be *judging* the observed). Afterwards, these field notes are transcribed into observation protocols. If more than one observer was involved, field notes are taken independently, but notes can be consolidated into one protocol after discussions. Advantages of conducting observations include minimising the distance between the researcher and the researched, the potential discovery of topics that the researcher did not realise were relevant and gaining deeper insights into the real-world dimensions of the research problem at hand [18].

#### Semi-structured interviews

Hijmans & Kuyper describe qualitative interviews as *“an exchange with an informal character, a conversation with a goal”* [19]. Interviews are used to gain insights into a person’s subjective experiences, opinions and motivations – as opposed to facts or behaviours [13]. Interviews can be distinguished by the degree to which they are structured (i.e. a questionnaire), open (e.g. free conversation or autobiographical interviews) or semi-structured [2, 13]. Semi-structured interviews are characterized by open-ended questions and the use of an *interview guide (or topic guide/list)* in which the broad areas of interest, sometimes including sub-questions, are defined [19]. The pre-defined topics in the interview guide can be derived from the literature, previous research or a preliminary method of data collection, e.g. document study or observations. The topic list is usually adapted and improved at the start of the data collection process as the interviewer learns more about the field [20]. Across interviews the focus on the different (blocks of) questions may differ and some questions may be skipped altogether (e.g. if the interviewee is not able or willing to answer the questions or for concerns about the total length of the interview) [20]. Qualitative interviews are usually not conducted in written format as it impedes on the interactive component of the method [20]. In comparison to written surveys, qualitative interviews have the advantage of being interactive and allowing for unexpected topics to emerge and to be taken up by the researcher. This can also help overcome a provider or researcher-centred bias often found in written surveys, which by nature, can only measure what is already known or expected to be of relevance to the researcher. Interviews can be audio- or video-taped; but sometimes it is only feasible or acceptable for the interviewer to take written notes [14, 16, 20].

#### Focus groups

Focus groups are group interviews to explore participants’ expertise and experiences, including explorations of how and why people behave in certain ways [1]. Focus groups usually consist of 6–8 people and are led by an experienced moderator following a topic guide or “script” [21]. They can involve an observer who takes note of the non-verbal aspects of the situation, possibly using an observation guide [21]. Depending on researchers’ and participants’ preferences, the discussions can be audio- or video-taped and transcribed afterwards [21]. Focus groups are useful for bringing together homogeneous (to a lesser extent heterogeneous) groups of participants with relevant expertise and experience on a given topic on which they can share detailed information [21]. Focus groups are a relatively easy, fast and inexpensive method to gain access to information on interactions in a given group, i.e. *“the sharing and comparing”* among participants [21]. Disadvantages include less control over the process and a lesser extent to which each individual may participate. Moreover, focus group moderators need experience, as do those tasked with the analysis of the resulting data. Focus groups can be less appropriate for discussing sensitive topics that participants might be reluctant to disclose in a group setting [13]. Moreover, attention must be paid to the emergence of “groupthink” as well as possible power dynamics within the group, e.g. when patients are awed or intimidated by health professionals.

#### Choosing the “right” method

As explained above, the school of thought underlying qualitative research assumes no objective hierarchy of evidence and methods. This means that each choice of single or combined methods has to be based on the research question that needs to be answered and a critical assessment with regard to whether or to what extent the chosen method can accomplish this – i.e. the “fit” between question and method [14]. It is necessary for these decisions to be documented when they are being made, and to be critically discussed when reporting methods and results.

**Fig. 2:**
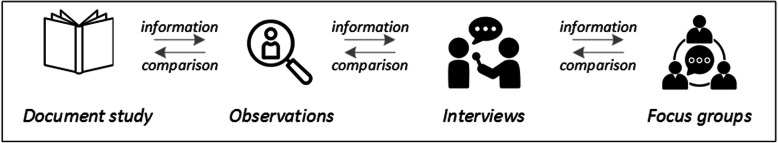
Possible combination of data collection methods

Let us assume that our research aim is to examine the (clinical) processes around acute endovascular treatment (EVT), from the patient’s arrival at the emergency room to recanalization, with the aim to identify possible causes for delay and/or other causes for sub-optimal treatment outcome. As a first step, we could conduct a document study of the relevant standard operating procedures (SOPs) for this phase of care – are they up-to-date and in line with current guidelines? Do they contain any mistakes, irregularities or uncertainties that could cause delays or other problems? Regardless of the answers to these questions, the results have to be interpreted based on what they are: a written outline of what care processes in this hospital *should* look like. If we want to know what they actually look like in practice, we can conduct observations of the processes described in the SOPs. These results can (and should) be analysed in themselves, but also in comparison to the results of the document analysis, especially as regards relevant discrepancies. Do the SOPs outline specific tests for which no equipment can be observed or tasks to be performed by specialized nurses who are not present during the observation? It might also be possible that the written SOP is outdated, but the actual care provided is in line with current best practice. In order to find out why these discrepancies exist, it can be useful to conduct interviews. Are the physicians simply not aware of the SOPs (because their existence is limited to the hospital’s intranet) or do they actively disagree with them or does the infrastructure make it impossible to provide the care as described? Another rationale for adding interviews is that some situations (or all of their possible variations for different patient groups or the day, night or weekend shift) cannot practically or ethically be observed. In this case, it is possible to ask those involved to report on their actions – being aware that this is not the same as the actual observation. A senior physician’s or hospital manager’s description of certain situations might differ from a nurse’s or junior physician’s one, maybe because they intentionally misrepresent facts or maybe because different aspects of the process are visible or important to them. In some cases, it can also be relevant to consider to whom the interviewee is disclosing this information – someone they trust, someone they are otherwise not connected to, or someone they suspect or are aware of being in a potentially “dangerous” power relationship to them. Lastly, a focus group could be conducted with representatives of the relevant professional groups to explore how and why exactly they provide care around EVT. The discussion might reveal discrepancies (between SOPs and actual care or between different physicians) and motivations to the researchers as well as to the focus group members that they might not have been aware of themselves. For the focus group to deliver relevant information, attention has to be paid to its composition and conduct, for example, to make sure that all participants feel safe to disclose sensitive or potentially problematic information or that the discussion is not dominated by (senior) physicians only. The resulting combination of data collection methods is shown in Fig. 2.

*Attributions for icons: “Book” by Serhii Smirnov, “Interview” by Adrien Coquet, FR, “Magnifying Glass” by anggun, ID, “Business communication” by Vectors Market; all from the Noun Project*

The combination of multiple data source as described for this example can be referred to as “triangulation”, in which multiple measurements are carried out from different angles to achieve a more comprehensive understanding of the phenomenon under study [22, 23].

### Data analysis

**Fig. 3:**
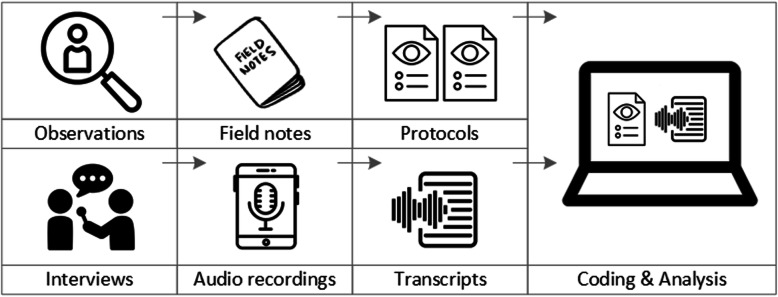
From data collection to data analysis

To analyse the data collected through observations, interviews and focus groups these need to be transcribed into protocols and transcripts (see Fig. 3). Interviews and focus groups can be transcribed *verbatim*, with or without annotations for behaviour (e.g. laughing, crying, pausing) and with or without phonetic transcription of dialects and filler words, depending on what is expected or known to be relevant for the analysis. In the next step, the protocols and transcripts are *coded*, that is, marked (or tagged, labelled) with one or more short descriptors of the content of a sentence or paragraph [2, 15, 23]. Jansen describes coding as *“connecting the raw data with “theoretical” terms”* [20]. In a more practical sense, coding makes raw data sortable. This makes it possible to extract and examine all segments describing, say, a tele-neurology consultation from multiple data sources (e.g. SOPs, emergency room observations, staff and patient interview). In a process of synthesis and abstraction, the codes are then grouped, summarised and/or categorised [15, 20]. The end product of the coding or analysis process is a descriptive theory of the behavioural pattern under investigation [20]. The coding process is performed using qualitative data management software, the most common ones being InVivo, MaxQDA and Atlas.ti. It should be noted that these are data *management* tools which support the *analysis* performed by the researcher(s) [14].

*Attributions for icons: see* Fig. 2*, also “Speech to text” by Trevor Dsouza, “Field Notes” by Mike O’Brien, US, “Voice Record” by ProSymbols, US, “Inspection” by Made, AU, and “Cloud” by Graphic Tigers; all from the Noun Project*

## How to report qualitative research?

Protocols of qualitative research can be published separately and in advance of the study results. However, the aim is not the same as in RCT protocols, i.e. to pre-define and set in stone the research questions and primary or secondary endpoints. Rather, it is a way to describe the research methods in detail, which might not be possible in the results paper given journals’ word limits. Qualitative research papers are usually longer than their quantitative counterparts to allow for deep understanding and so-called “thick description”. In the methods section, the focus is on transparency of the methods used, including why, how and by whom they were implemented in the specific study setting, so as to enable a discussion of whether and how this may have influenced data collection, analysis and interpretation. The results section usually starts with a paragraph outlining the main findings, followed by more detailed descriptions of, for example, the commonalities, discrepancies or exceptions per category [20]. Here it is important to support main findings by relevant quotations, which may add information, context, emphasis or real-life examples [20, 23]. It is subject to debate in the field whether it is relevant to state the exact number or percentage of respondents supporting a certain statement (e.g. “Five interviewees expressed negative feelings towards XYZ”) [21].

## How to combine qualitative with quantitative research?

**Fig. 4:**
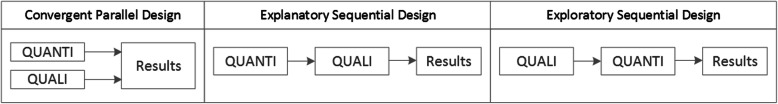
Three common mixed methods designs

Qualitative methods can be combined with other methods in multi- or mixed methods designs, which *“[employ] two or more different methods [ …] within the same study or research program rather than confining the research to one single method”* [24]. Reasons for combining methods can be diverse, including *triangulation* for corroboration of findings, *complementarity* for illustration and clarification of results, *expansion* to extend the breadth and range of the study, *explanation* of (unexpected) results generated with one method with the help of another, or *offsetting* the weakness of one method with the strength of another [1, 17, 24–26]. The resulting designs can be classified according to when, why and how the different quantitative and/or qualitative data strands are combined. The three most common types of mixed method designs are the *convergent parallel design*, the *explanatory sequential design* and the *exploratory sequential design.* The designs with examples are shown in Fig. 4.

In the convergent parallel design, a qualitative study is conducted in parallel to and independently of a quantitative study, and the results of both studies are compared and combined at the stage of interpretation of results. Using the above example of EVT provision, this could entail setting up a quantitative EVT registry to measure process times and patient outcomes in parallel to conducting the qualitative research outlined above, and then comparing results. Amongst other things, this would make it possible to assess whether interview respondents’ subjective impressions of patients receiving good care match modified Rankin Scores at follow-up, or whether observed delays in care provision are exceptions or the rule when compared to door-to-needle times as documented in the registry. In the explanatory sequential design, a quantitative study is carried out first, followed by a qualitative study to help explain the results from the quantitative study. This would be an appropriate design if the registry alone had revealed relevant delays in door-to-needle times and the qualitative study would be used to understand where and why these occurred, and how they could be improved. In the exploratory design, the qualitative study is carried out first and its results help informing and building the quantitative study in the next step [26]. If the qualitative study around EVT provision had shown a high level of dissatisfaction among the staff members involved, a quantitative questionnaire investigating staff satisfaction could be set up in the next step, informed by the qualitative study on which topics dissatisfaction had been expressed. Amongst other things, the questionnaire design would make it possible to widen the reach of the research to more respondents from different (types of) hospitals, regions, countries or settings, and to conduct sub-group analyses for different professional groups.

## How to assess qualitative research?

A variety of assessment criteria and lists have been developed for qualitative research, ranging in their focus and comprehensiveness [14, 17, 27]. However, none of these has been elevated to the “gold standard” in the field. In the following, we therefore focus on a set of commonly used assessment criteria that, from a practical standpoint, a researcher can look for when assessing a qualitative research report or paper.

### Checklists

Assessors should check the authors’ use of and adherence to the relevant reporting checklists (e.g. Standards for Reporting Qualitative Research (SRQR)) to make sure all items that are relevant for this type of research are addressed [23, 28]. Discussions of quantitative measures in addition to or instead of these qualitative measures can be a sign of lower quality of the research (paper). Providing and adhering to a checklist for qualitative research contributes to an important quality criterion for qualitative research, namely transparency [15, 17, 23].

### Reflexivity

While methodological transparency and complete reporting is relevant for all types of research, some additional criteria must be taken into account for qualitative research. This includes what is called reflexivity, i.e. sensitivity to the relationship between the researcher and the researched, including how contact was established and maintained, or the background and experience of the researcher(s) involved in data collection and analysis. Depending on the research question and population to be researched this can be limited to professional experience, but it may also include gender, age or ethnicity [17, 27]. These details are relevant because in qualitative research, as opposed to quantitative research, the researcher as a person cannot be isolated from the research process [23]. It may influence the conversation when an interviewed patient speaks to an interviewer who is a physician, or when an interviewee is asked to discuss a gynaecological procedure with a male interviewer, and therefore the reader must be made aware of these details [19].

### Sampling and saturation

The aim of qualitative sampling is for all *variants* of the objects of observation that are deemed relevant for the study to be present in the sample “*to see the issue and its meanings from as many angles as possible”* [1, 16, 19, 20, 27]*,* and to ensure *“information-richness* [15]. An iterative sampling approach is advised, in which data collection (e.g. five interviews) is followed by data analysis, followed by more data collection to find variants that are lacking in the current sample. This process continues until no new (relevant) information can be found and further sampling becomes redundant – which is called *saturation* [1, 15]*.* In other words: qualitative data collection finds its end point not *a priori*, but when the research team determines that saturation has been reached [29, 30].

This is also the reason why most qualitative studies use *deliberate* instead of random sampling strategies. This is generally referred to as “*purposive sampling”*, in which researchers pre-define which types of participants or cases they need to include so as to cover all variations that are expected to be of relevance, based on the literature, previous experience or theory (i.e. theoretical sampling) [14, 20]. Other types of purposive sampling include (but are not limited to) maximum variation sampling, critical case sampling or extreme or deviant case sampling [2]. In the above EVT example, a purposive sample could include all relevant professional groups and/or all relevant stakeholders (patients, relatives) and/or all relevant times of observation (day, night and weekend shift).

Assessors of qualitative research should check whether the considerations underlying the sampling strategy were sound and whether or how researchers tried to adapt and improve their strategies in stepwise or cyclical approaches between data collection and analysis to achieve saturation [14].

### Piloting

Good qualitative research is iterative in nature, i.e. it goes back and forth between data collection and analysis, revising and improving the approach where necessary. One example of this are pilot interviews, where different aspects of the interview (especially the interview guide, but also, for example, the site of the interview or whether the interview can be audio-recorded) are tested with a small number of respondents, evaluated and revised [19]. In doing so, the interviewer learns which wording or types of questions work best, or which is the best length of an interview with patients who have trouble concentrating for an extended time. Of course, the same reasoning applies to observations or focus groups which can also be piloted.

### Co-coding

Ideally, coding should be performed by at least two researchers, especially at the beginning of the coding process when a common approach must be defined, including the establishment of a useful coding list (or tree), and when a common meaning of individual codes must be established [23]. An initial sub-set or all transcripts can be coded independently by the coders and then compared and consolidated after regular discussions in the research team. This is to make sure that codes are applied consistently to the research data.

### Member checking

Member checking, also called *respondent validation*, refers to the practice of checking back with study respondents to see if the research is in line with their views [14, 27]. This can happen after data collection or analysis or when first results are available [23]. For example, interviewees can be provided with (summaries of) their transcripts and asked whether they believe this to be a complete representation of their views or whether they would like to clarify or elaborate on their responses [17]. Respondents’ feedback on these issues then becomes part of the data collection and analysis [27].

### Stakeholder involvement

In those niches where qualitative approaches have been able to evolve and grow, a new trend has seen the inclusion of patients and their representatives not only as study participants (i.e. “members”, see above) but as consultants to and active participants in the broader research process [31–33]. The underlying assumption is that patients and other stakeholders hold unique perspectives and experiences that add value beyond their own single story, making the research more relevant and beneficial to researchers, study participants and (future) patients alike [34, 35]. Using the example of patients on or nearing dialysis, a recent scoping review found that 80% of clinical research did not address the top 10 research priorities identified by patients and caregivers [32, 36]. In this sense, the involvement of the relevant stakeholders, especially patients and relatives, is increasingly being seen as a quality indicator in and of itself.

## How *not* to assess qualitative research

The above overview does not include certain items that are routine in assessments of quantitative research. What follows is a non-exhaustive, non-representative, experience-based list of the quantitative criteria often applied to the assessment of qualitative research, as well as an explanation of the limited usefulness of these endeavours.

### Protocol adherence

Given the openness and flexibility of qualitative research, it should not be assessed by how well it adheres to pre-determined and fixed strategies – in other words: its rigidity. Instead, the assessor should look for signs of adaptation and refinement based on lessons learned from earlier steps in the research process.

### Sample size

For the reasons explained above, qualitative research does not require specific sample sizes, nor does it require that the sample size be determined a priori [1, 14, 27, 37–39]. Sample size can only be a useful quality indicator when related to the research purpose, the chosen methodology and the composition of the sample, i.e. who was included and why.

### Randomisation

While some authors argue that randomisation *can* be used in qualitative research, this is not commonly the case, as neither its feasibility nor its necessity or usefulness has been convincingly established for qualitative research [13, 27]. Relevant disadvantages include the negative impact of a too large sample size as well as the possibility (or probability) of selecting “*quiet, uncooperative or inarticulate individuals*” [17]. Qualitative studies do not use control groups, either.

### Interrater reliability, variability and other “objectivity checks”

The concept of “interrater reliability” is sometimes used in qualitative research to assess to which extent the coding approach overlaps between the two co-coders. However, it is not clear what this measure tells us about the quality of the analysis [23]. This means that these scores *can* be included in qualitative research reports, preferably with some additional information on what the score means for the analysis, but it is not a requirement. Relatedly, it is not relevant for the quality or “objectivity” of qualitative research to separate those who recruited the study participants and collected and analysed the data. Experiences even show that it might be *better* to have the same person or team perform all of these tasks [20]. First, when researchers introduce themselves during recruitment this can enhance trust when the interview takes place days or weeks later with the same researcher. Second, when the audio-recording is transcribed for analysis, the researcher conducting the interviews will usually remember the interviewee and the specific interview situation during data analysis. This might be helpful in providing additional context information for interpretation of data, e.g. on whether something might have been meant as a joke [18].

### Not being quantitative research

Being qualitative research instead of quantitative research should not be used as an assessment criterion if it is used irrespectively of the research problem at hand. Similarly, qualitative research should not be required to be combined with quantitative research per se – unless mixed methods research is judged as inherently better than single-method research. In this case, the same criterion should be applied for quantitative studies without a qualitative component.

## Conclusion

The main take-away points of this paper are summarised in Table 1. We aimed to show that, if conducted well, qualitative research can answer specific research questions that cannot to be adequately answered using (only) quantitative designs. Seeing qualitative and quantitative methods as equal will help us become more aware and critical of the “fit” between the research problem and our chosen methods: I *can* conduct an RCT to determine the reasons for transportation delays of acute stroke patients – but *should* I? It also provides us with a greater range of tools to tackle a greater range of research problems more appropriately and successfully, filling in the blind spots on one half of the methodological spectrum to better address the whole complexity of neurological research and practice.

**Table 1:** Take-away-points

**Types of research problems**	**Data collection**	**Data analysis**
• Assessing complex multi-component interventions or systems (of change) • What works for whom when, how and why? • Focussing on intervention improvement	• Document study • Observations (participant or non-participant) • Interviews (especially semi-structured) • Focus groups	• Transcription of audio-recordings and field notes into transcripts and protocols • Coding of protocols • Using qualitative data management software
**Mixed and multi-method**	**How to assess**	**How not to assess**
• Combinations of quantitative and/or qualitative methods, e.g.: • *convergent parallel*: quali and quanti in parallel • *explanatory sequential*: quanti followed by quali • *exploratory sequential*: quali followed by quanti	• Checklists • Reflexivity • Sampling strategies • Piloting • Co-coding • Member checking • Stakeholder involvement	• Protocol adherence • Sample size • Randomization • Interrater reliability, variability and other “objectivity checks” • Not being quantitative research

## Data Availability

Not applicable.

## Abbreviations

EVT: Endovascular treatment
RCT: Randomised Controlled Trial
SOP: Standard Operating Procedure
SRQR: Standards for Reporting Qualitative Research
